# Parasites of small Indian mongoose, *Herpestes auropunctatus*, on St. Kitts, West Indies

**DOI:** 10.1007/s00436-018-5773-2

**Published:** 2018-01-30

**Authors:** Trista Cheng, Brandon Halper, Jennifer Siebert, Luis Cruz-Martinez, Aspinas Chapwanya, Patrick Kelly, Jennifer K. Ketzis, Jeffrey Vessell, Liza Köster, Chaoqun Yao

**Affiliations:** 1 Department of Biomedical Sciences, Ross University School of Veterinary Medicine, West Indies, Saint Kitts and Nevis; 2 One Health Center for Zoonosis and Tropical Veterinary Medicine, Ross University School of Veterinary Medicine, West Indies, Saint Kitts and Nevis; 3 Department of Clinical Sciences, Ross University School of Veterinary Medicine, West Indies, Saint Kitts and Nevis; 4 Present Address: Davies Vet Specialists, Manor Farm Business Park, Hingham Gobion, Hertfordshire, SG5 3HR UK

**Keywords:** Invasive species, Fleas, Small Indian mongoose, St. Kitts, *Toxoplasma gondii*, *Ctenocephalides felis*

## Abstract

*Herpestes auropunctatus*, the small Indian mongoose, is an invasive omnivore introduced to the Caribbean, including the island of St. Kitts over 150 years ago. It has played a role in changing native fauna and can carry zoonotic pathogens of public health importance. The aim of the current study was to estimate the prevalence of parasites harbored by mongooses. In total, 87 mongooses trapped from April to July 2015 were examined for parasites using (1) hair plucks (*N* = 79), ear swabs (*N* = 79), and general coat and skin examination (*N* = 87) for mites, ticks, lice, and fleas; (2) dissection of the trachea, bronchi, and lungs for lungworms and flukes (*N* = 76); (3) a double centrifugation fecal flotation method for parasites of the gastrointestinal tract (*N* = 75); and (4) PCR of heart homogenates for *Toxoplasma gondii* (*N* = 60). The only ectoparasite seen was *Ctenocephalides felis* (79.3%; 69/87), with most mongooses having > 10 fleas (based on a subjective assessment) but insufficient numbers to result in signs of pruritus or anemia. On fecal flotation, coccidial oocysts were found with a prevalence of 69.3% (52/75). Neither *T*. *gondii*, lungworm, nor fluke infections were detected with the methods used. The high number of *C. felis*-infested mongooses and the infestation level of the individual mongooses suggest that they could serve as a reservoir for these potential vectors of pathogens. No evidence was found to support that mongooses are a component of *T*. *gondii* cycles on St. Kitts, although this finding needs to be confirmed with a larger sample size from other geographic locations.

**Electronic supplementary material:**

The online version of this article (10.1007/s00436-018-5773-2) contains supplementary material, which is available to authorized users.

## Introduction

The small Indian mongoose, *Herpestes auropunctatus*, was introduced on several Caribbean islands in the West Indies to control rats, which were devastating sugar cane plantations, and to control poisonous snakes (Nellis and Everard 1983; Barun et al. 2011). This invasive species is now established on 33 Caribbean islands (Barun et al. 2011) with the extinction of several native wildlife attributed to them (e.g., the Red-bellied Racer (*Alsophis rufiventris*), the skink (*Mabuya* spp.), the Giant Ditch Frog (*Leptodactylus fallax*), and the ground lizard (*Ameiva erythrocephala*)) (Cooper et al. 2011). In addition to being potentially destructive to native wildlife, mongooses can be infected with pathogens of zoonotic potential such as *Salmonella* spp. (Miller et al. 2015), *Leptospira* spp. (Everard et al. 1976), rabies (Zieger et al. 2014; Berentsen et al. 2015), zoonotic parasites, and vectors of zoonotic pathogens. Presence of these zoonoses is of particular importance since, despite their shyness of humans, mongooses frequently visit fruit and vegetable gardens and readily inhabit areas of high human activity (Jessica and Desley 2005). However, while mongooses occur on many of the Caribbean islands, there are few studies on the prevalence of zoonotic organisms, particularly zoonotic parasites and vectors of zoonoses.

Of the zoonotic parasites known to occur in mongooses, *Toxoplasma gondii* is of particular concern and has been documented in mongooses in Saudi Arabia and Grenada (Dakhil and Morsy 1996; Choudhary et al. 2013). It involves mice in its life cycle and mongooses are known to consume mice. On some Caribbean islands, pigs (with *T*. *gondii* seroprevalence of 48% on St. Kitts) are allowed to roam free or are tethered in areas of vegetation (GEF Small Grants Programme 2017; Hamilton et al. 2015). These pigs could consume carcasses of infected mongooses (Abernethy et al. 2016). Therefore, mongooses could indirectly contribute to human exposure to *T*. *gondii*. Although consumption of mongoose carcasses by cats was not documented by Abernethy et al. (2016), scavenging behavior is influenced by season and location (Turner et al. 2017); therefore, mongooses could be in the food chain of feral cats and might contribute to the high prevalence of *T*. *gondii* in cats (up to 84% seropositive) which has been documented on some of the Caribbean islands (Moura et al. 2007; Dubey et al. 2009; Hamilton et al. 2015). In addition to *T*. *gondii*, mongooses are a potential reservoir for *Leishmania donovani*, a protozoan parasite causing human visceral leishmaniasis, with the Egyptian mongoose, *Herpestes ichneumon*, found to be infected in Sudan (Elnaiem et al. 2001).

Mongooses also have been found to harbor the zoonotic ectoparasites *Pulex irritans* (the “human flea”) and *Notoedres felis* (mange mite) as well as ectoparasites associated with disease transmission to humans and domestic animals (e.g., *Ctenocephalides felis* and *Amblyomma variegatum*) (Nellis and Everard 1983; Corn et al. 1994; Corn et al. 2009; Townsend and Powers 2014). A better understanding of the role of mongooses in the life cycle of *T*. *gondii* as well as other parasites and pathogen vectors is useful for governments and health organizations developing policies on controlling human and animal diseases in the region.

The purpose of the current study was to estimate the prevalence of parasites, with a focus on *T*. *gondii* and ectoparasites, in the small Indian mongoose population on St. Kitts, located in the Lesser Antilles in the Caribbean (17° 15′ N, 62° 45′ W), with an area of 179 km^2^ (Kittitian-Government 2017).

## Materials and methods

### Ethics

Animal care and use were in accordance with the recommendations in the Guide for the Care and Use of Laboratory Animals of the US National Institutes of Health. All animal work was performed under a protocol (# 15-2-007) approved by the Institutional Animal Care and Use Committee (IACUC) of Ross University School of Veterinary Medicine.

### Sample size and study sites

The mongoose population size on St. Kitts is unknown; using the lower population density from other islands, it could be over 45,000, which is high when compared to a human population of slightly less than 57,000. The mongooses reside throughout the island in arid beach areas, rural, urban and residential areas, and in more arid areas at higher altitudes (> 800 m) inland. The mongooses used were trapped and euthanized for an unrelated study to determine if they were rabies free. To maximize the use of the mongooses, additional samples, including those used in this study, were collected. The number of mongooses and study sites were dictated by the rabies study and not all mongooses trapped were available for parasitological examination. However, with an estimated parasite prevalence of 5% and population estimate of > 10,000, 73 mongooses were the required minimum sample size to detect infection with a 95% confidence level (CL) (EpiTools epidemiological calculators 2017). The study sites corresponded to three beach areas (17° 13′ 38′′ N, 62° 38′ 48′′ W) and one residential area (17° 18′ 24′′ N, 62° 41′ 29′′ W) on St. Kitts. The sites were selected based on nearness to people and feral cat colonies, and because these sites provide suitable habitat for mongooses. They were approximately 0.5 km^2^ in size. Estimate of area of the combined study sites compared to the area of the island was 1:358.

### Animal trapping and euthanasia

The 87 mongooses that were included in this study were trapped between April and July 2015 using a commercial box trap (19 × 19 × 48 cm; Tomahawk Live Trap, WI, USA) and canned tuna as bait. The traps were set up at dawn three times per week (Mondays, Wednesdays, and Fridays) and placed in shaded areas of vegetation adjacent to the beaches or near houses. Traps were set for a maximum of 2 h with any mongooses caught during this time transported to facilities at Ross University School of Veterinary Medicine for euthanasia. The mongooses were anesthetized using a manual vaporizer (JorVet Vapor Wand, Jorgensen laboratories, Loveland, CO, USA) and isoflurane (Sigma, St. Louis, CO, USA); once anesthesia was induced, the mongooses were kept in a deep anesthetic plane (3.5% isoflurane) using a portable anesthesia machine. The mongooses were then euthanized by an intracardiac injection of potassium chloride (1–2 mmol/kg) with necropsy and inspection for parasites performed immediately after euthanasia (Zieger et al. 2014).

### Inspection for ectoparasites

The coat and skin of each mongooses was inspected visually for any signs of mite, lice, flea, and tick infestations. One to five fleas were removed from any infested mongoose and any other ectoparasites seen were removed and placed in 70% ethanol for identification. Given that many fleas were seen jumping off of the mongooses, a subjective assessment on flea infestation levels was made. For fleas found, keys from Pratt and Stark (1973) were used for species identification. Hair plucks (from back legs, body, and head; minimum of 20 hairs per location) and skin scrapes (from the same region as the plucks) were mixed with mineral oil and placed on glass slides or petri dishes. Ear swabs were collected for ear mite examination with the swabs teased apart in a petri dish. All samples were examined for mites and lice (eggs, immature stages, and adults) at × 20 to × 100 magnification using a stereo microscope.

### Inspection for lungworms and flukes in the respiratory system

Lungs were examined for nodules by visual inspection and palpation with a gloved hand. The trachea, bronchi, bronchioles, and lungs were dissected with fine-tipped scissors and examined for the presence of nematodes using a hand-held illuminated magnifying glass (× 2 magnification with 4X spot, Fisher Scientific, Hampton, NH, USA).

### Detection of parasites in the gastrointestinal tract

The gastrointestinal (GI) tract was opened longitudinally and the contents collected and submitted to the Ross University Veterinary Diagnostic Laboratory. Any macroscopic GI parasites were noted and recorded when the contents were collected. The contents were analyzed using double centrifugation with Sheather’s sugar flotation solution (specific gravity 1.27–1.28) and examined for GI parasites, parasite eggs, cysts, and oocysts under × 100 and × 400 magnification (Zajac and Conboy 2012). Keys from Phillips (1990) were used to identify mites.

### Molecular detection of *T*. *gondii*

#### Cardiac muscle digestion and DNA extraction

It was recently shown that detecting *T*. *gondii* DNA in cardiac tissues of domestic animals including pigs, sheep, and goats was correlated to a widely used serological test (Hamilton et al. 2015). So heart tissues were used for detection and phenotyping of *T*. *gondii*. Each heart (*N* = 76; average weight of 2.49 ± 1.66 g) was cut into small pieces with scissors and placed in a 50 ml conical tube with 40 ml of the 1% pepsin-1% HCl solution (Sigma) as previously described (Yao et al. 1997). The sample was incubated at 37 °C for at least 2 h with constant rocking. Afterwards, the slurry was poured through a strainer into a 50 ml conical tube and centrifuged for 10 min at 2000×*g* at 4 °C. The pellet was washed twice in phosphate-buffered saline (pH 7.4, Sigma) and centrifuged, as described, between each washing. The heart homogenate pellet of the last centrifugation was frozen (−20 °C) until DNA extraction. Prior to DNA extraction, the homogenate of each heart was thawed at room temperature and DNA was extracted from the entire homogenate using a DNeasy Blood and Tissue Kit (QIAGEN, Hilden, Germany) following the manufacturer’s protocol. The Tecan Infinite M200 Pro (Tecan, Mannedorf, Switzerland) was used to monitor DNA quality and quantity. DNA was stored at −20 °C until further use.

#### Detection of *T*. *gondii* by PCR

All PCR was performed using a thermal cycler (Mastercycler Nexus Gradient, Eppendorf, Hauppauge, NY, USA). PCR reagent was HotStart Taq Plus 2 × Master Mix (QIAGEN). Primers Tox-9F (5′AGGAGAGATATCGGGACTGTAG3′) and Tox-11R (5′GCGTCGTCTCGTCTAGATCG3′) were synthesized by IDT (Coralville, IA, USA). These primers targeted the 529-bp repeat element of *T*. *gondii* with an expected product of 163 bp (Homan et al. 2000; Opsteegh et al. 2010). PCR mix (20 μl) was used for each reaction with a final concentration of each forward and reverse primer at 0.5 μM. PCR was performed as previously described (Opsteegh et al. 2010; Hamilton et al. 2015): 95 °C 2 min followed by 45 cycles of 95 °C 30 s, 58 °C 30 s, and 72 °C 1 min with a final extension of 72 °C for 10 min. Due to variation in DNA concentration in the samples, various volumes of DNA samples were used. For samples with DNA concentration ≥ 200, ≥ 100, ≥ 50, or ≥ 20 ng/μl, 1, 2, 4, and 9.8 μl of DNA samples were used in each PCR reaction, respectively. The samples with DNA concentration lower than 20 ng/μl were not tested. For each batch of PCR reactions, a positive and a negative control were included. Positive control DNA was the genomic DNA with a concentration of 27.4 fg/μl of the RH strain (genotype I) of *T*. *gondii* organisms isolated from animal tissues that had been subjected to pepsin-HCl digestion prior to DNA extraction. Negative control was molecular grade water supplied with PCR reagents (QIAGEN). The amplicons were visualized via electrophoresis on a 1.5% agarose gel using a 100-bp ladder (Invitrogen, Carlsbad, CA, USA) as a size marker.

## Results

### Ectoparasites

No obvious external signs of mite infestations (e.g., skin lesions, hair loss, salt and pepper hair appearance) were seen in any of the mongooses. No stages of mites or lice, including eggs, were seen in the hair plucks, skin scrapes, and ear swabs (*N* = 79). No lice or ticks were found on any of the mongooses during visual examination. Fleas were recovered from 69 of 87 mongooses (79.3%) (95% CI 70.8–87.8%). All fleas were identified as *Ctenocephalides felis*. All of the mongooses with fleas appeared to have at least ten fleas with several having heavier infestations (e.g., 25–50), although exact numbers were not counted.

### Lungworms and flukes

No parasites (nematodes or flukes) were grossly visible or detected at low magnification in the trachea, bronchi, bronchioles, and lungs of all animals examined (*N* = 76).

### GI parasites

Fifty-two of 75 (69.3%, 95% CI 58.9–79.8%) samples were positive for coccidial oocysts. No other GI parasites were seen during gross inspection of the GI contents or in the centrifuged and floated contents. Free-living mites and mite eggs were present in three animals (4.0%, 95% CI 0–8.4%).

### *Toxoplasma gondii* in the heart

Sixty of the 76 samples had a DNA concentration ≥ 20 ng/μl and were analyzed via PCR using the volumes summarized in the supplementary Table 1. An average of 282 to 339 ng total DNA was used in each PCR. Two of 60 samples were initially PCR positive for *T*. *gondii*, but were tested negative when the PCR was repeated, twice, and were thus considered as negative.

## Discussion

The scope of our study focused on estimating the prevalence of parasites, especially *T*. *gondii* and ectoparasites, in the small Indian mongooses on St. Kitts focusing on mongooses residing in beach areas and more arid areas, including residential areas. Results indicate that mongooses on St. Kitts harbor coccidia. Coccidial oocysts were seen in the GI tract of 69.3% animal examined. They are very likely to be *Isospora herpestei* based on host species according to Levine et al. (1975), although there was no attempt to identify them. Therefore, these coccidia are not a zoonotic concern. The mongooses on St. Kitts harbor *C*. *felis* with a 79.3% prevalence, which can be vectors for zoonotic pathogens.

Studies on Antigua, Grenada, St. Croix, St. John, Trinidad, and other Caribbean islands have indicated that mongooses serve as a reservoir for *C*. *felis* (Nellis and Everard 1983). This is in agreement with the findings in this study, although the prevalence in this study, as well as the infection intensity, was higher than that documented in recent studies on other islands where mongooses were found to be infested typically with only 1–2 fleas (Corn et al. 2009; Townsend and Powers 2014). Other flea species, ticks (*Amblyomma* spp. and *Carios* spp.), non-parasitic mites, and fly larvae, which have been recovered from small Indian mongooses on other islands, were not seen in this study (Nellis and Everard 1983; Corn et al. 1994; Corn et al. 2009; Townsend and Powers 2014). Of these ectoparasites, the most important from a human and domestic animal perspective are *C*. *felis* and *A*. *variegatum*. *Ctenocephalides felis*, the common dog and cat flea, can transmit *Rickettsia felis* to cats and people (Parola 2011). A study on St. Kitts indicated that 19% of *C*. *felis* harvested from cats were positive for *R*. *felis* (Kelly et al. 2010b). The role of *C*. *felis* from mongooses in transmission of *R*. *felis* is not known. Further studies are required to determine if *C*. *felis* is transmitted between feral cats and mongooses on St. Kitts, if *R*. *felis* prevalence in the fleas on mongooses is similar to that in fleas on feral cats, and if mongooses serve as a reservoir for *R*. *felis* infection in people. The cat flea is also a vector of *Bartonella henselae* and *B*. *clarridgeiae*, which are agents of cat-scratch disease in people and known to occur in feral cats on St. Kitts (Kelly et al. 2010c). *Amblyomma variegatum* is an important vector of *Dermatophilus* spp. (dermatophilosis) on St. Kitts and *Dermatophilus* spp. and *Ehrlichia ruminantium* (heartwater) on other Caribbean islands. These are debilitating cattle diseases that impact the food self-sufficiency of the islands (Corn et al. 2009). *Amblyomma variegatum* on St. Kitts also have been noted to be infected with *Rickettsia africae*, the causative agent of tick-bite fever in people (Kelly et al. 2010a). Not detecting *A*. *variegatum* on the mongooses on St. Kitts might be due to the trapping locations which were not in cattle grazing areas and indicates that coastal mongoose colonies could likely be excluded from *A*. *variegatum* control programs on St. Kitts that include wildlife hosts. Not detecting diversity in ectoparasites seen in this study might be related to the trapping sites. Barre et al. (1988) noted differences in tick infestation levels based on topography and rainfall; therefore, mongooses on St. Kitts might also have higher infestations and more diversity in non-coastal areas (Barre et al. 1988).

As far as GI parasites are concerned, the lack of nematodes differs from earlier reports. Nellis and Everard (1983) found three genera of nematodes: *Physaloptera*, *Capillaria*, and *Skrjabinocapillaria*. The lack of cestodes and trematodes are in agreement with other reports (Nellis and Everard 1983). It must be noted, however, that since specific tests for parasites such as trichomonads and *Giardia* spp. were not performed, their presence cannot be excluded.

Not detecting *T*. *gondii* in the mongooses was a surprising result given its occurrence in mongooses on other Caribbean islands and the high seroprevalence in feral cats and domestic animals on St. Kitts. Hamilton et al. (2015), also using heart homogenates and the same PCR methods, detected *T*. *gondii* in pigs, sheep, and goats on St. Kitts with a prevalence of 21, 16, and 23%, respectively (Hamilton et al. 2015). T*oxoplasma gondii* also has been found to be widespread in the feral cat population on the island with 84.9% (90/106) seropositive for *T*. *gondii* antibodies based on a modified agglutination test (MAT) in 2005–2006 and 73.9% (71/96) positive by MAT in 2005–2006 (Moura et al. 2007; Dubey et al. 2009). Studies on other Caribbean islands have shown that mongooses in Grenada were 29.7% (27/91) seropositive by MAT and 16.0% (4/25) by bio-assay for *T*. *gondii* in 2011 and 2012 (Choudhary et al. 2013). Although serologic testing was more sensitive than the molecular technique, the two methods showed a moderate correlation (*k* = 0.5204) for detecting *T*. *gondii* infection (Hamilton et al. 2015). In this study, molecular detection was used instead of serologic testing so that samples would have been available for genotyping. In light of the findings of *T*. *gondii* in mongooses from Grenada, the known high prevalence of the organism on St. Kitts and that the parasite can probably infect all warm blooded animals (birds and mammals), we suspect that not finding infected mongooses is most likely due to our small sample size, the weight per sample, and the relatively small area from which the mongooses were trapped. Further studies using serologic testing and greater numbers of animals from more diverse locations are required to more precisely determine the status of *T*. *gondii* in the mongooses on St. Kitts. Instead of using PCR to detect the DNA of the parasite, a seroepidemiological study might be more suitable for this.

In summary, small Indian mongooses on St. Kitts were examined to estimate the prevalence of parasites with a focus on *T*. *gondii* and ectoparasites. With the methods applied, the only parasites found were *C*. *felis* and coccidia. Based on these findings, it does not appear that mongooses pose an appreciable risk for zoonotic parasite transmission on St. Kitts. However, further investigation is warranted particularly in regards to *T*. *gondii* and the role of *C*. *felis* on mongooses as potential vector of pathogens. Also, testing of mongooses in less arid areas of the island, particularly in areas with more livestock, should be performed to determine the influence of environment on infections and infestations.

## Electronic supplementary material

Supplementary Table 1 (DOCX 12 kb)
