# Supplementary material for: Parasites of small Indian mongoose, Herpestes auropunctatus, on St. Kitts, West Indies
Source: Parasitol Res. 2018 Jan 30;117(4):989–94. doi: 10.1007/s00436-018-5773-2 (PMC5978914; doi:10.1007/s00436-018-5773-2)
Supplement: Supplementary file 1 — (DOCX 12 kb) [file 436_2018_5773_MOESM1_ESM.docx]

Supplementary Table 1. Concentration and amount of DNA of cardiac samples from small Indian mongoose, *Herpestes auropunctatus*, used in PCR amplification of the 529-bp repeat element of *Toxoplasma gondii*.

| DNA concen-tration ng/µl | Number of samples | Average (Median) ng/µl | µl used in each PCR | Average DNA ng/PCR |
| --- | --- | --- | --- | --- |
| ≥20 | 17 | 32.5 (33.6) | 9.8 | 318.5 |
| ≥50 | 15 | 70.7 (69.5) | 4 | 282.8 |
| ≥100 | 17 | 150.8 (156.1) | 2 | 301.6 |
| ≥200 | 11 | 339.3 (282.2) | 1 | 339.3 |
| Total | 60 |  |  |  |
